# Abolition of a Rule in Medical Graduation

**Published:** 1843-01

**Authors:** 


ABOLITION OF A RULE IN MEDICAL GRADUATION.—WILLOUGHBY UNIUERSITY OF LAKE ERIE.

The readers of the "Western Journal of the Medical and Physical Sciences," will recollect our former notices of this medical institution, situate in the north-east corner, if it can be said to have corners, of our great western valley. The village where it is established, lies eighteen miles east of Cleveland, on the bank of the little river Chagrin, (Chaguin?). While in that region we spent an hour within the walls of the University edifice, and saw three of its professors. We are enabled to say, that it has a good germinal assemblage of the means of medical education, that its last class amounted to 57, and that its Faculty is decidedly more able, at the present, than any past time. But our chief object is not to make these facts known, seeing that most of our readers live beyond the region whence that school looks for pupils.

What we aim to disseminate, is the fact, communicated to us by one of the Professors and also set forth in their last circular, that they have abolished the regulation, which allowed a four years practice to be equal, in reference to graduation, to a course of lectures. We look upon this as a decided improvement in the economy of western schools, and hope to see it adopted by all.

Where or when this rule was first incorporated into the code of regulations of our schools, we shall not stop to inquire. The first we ever heard of it, was in Transylvania University, on the revival of its medical department in 1819. In the succeeding year it was adopted by the Medical College of Ohio; some years after by the Willoughby school; in 1835, by the Cincinnati College; in 1837, by the Medical Institute of Louisville; in 1840, by the Medical Department of Kemper College, St. Louis; and in 1842, by the

Medical Department of the University of St. Louis. Thus it has, as they successively came into existence, been made a feature of all the schools of the west.

We do not question the propriety of this regulation, at the time, more than twenty years ago, when it was promulgated by the schools of Lexington and Cincinnati; for, as they were the first in the west, it embraced a great number of physicians who had, from the distance and difficult access of the schools of Philadelphia, New York and Baltimore, never attended lectures any where; and it was, perhaps, sound policy (we use the word in reference to the dignity of the profession) to allow such of them, as would attend one course of lectures, the privilege of becoming candidates for graduation. But the rule should have been regarded as applicable *only* to those who were *then* in practice, and ought to have been abrogated at the end of the first four years; that is, as soon as all who had *entered* on the practice previous to the time of its promulgation, had had the opportunity of availing themselves of its benefits.

The mischiefs arising from its continuance, have, we cannot doubt, been very considerable. Many who would have attended one course of lectures, *before* engaging in practice, have been induced to postpone it, till, by standing before the community as practitioners for four years, they could, on a single course, become candidates; and to shorten this probationary period, it is unquestionably true, that many have engaged in the practical duties of the profession, after a very brief period of office study, and consequently with most limited and superficial acquirements; which too often, have been but slightly extended during those years. With some honorable exceptions, when such "young doctors" come to our schools, they have travelled on the narrow and devious path, quite out of the region of rudimental facts and principles. Anatomy, physiology, chemistry and pharmacy, of which they know but little, are so far behind the practical rules and recipes of physic, surgery and obstetrics, upon which their minds have been directed, that they cannot be "turned back." They are unwilling to admit, even to themselves, that they have been going forth into society, as practical physicians and surgeons, ignorant of the elementary truths to which they find the junior students attentive; and, therefore, (again, with some respectable exceptions,) present themselves for examination, with many radical and incurable defects. It may be said, if they are not *well* prepared, let them be rejected?

But is not this a heartless remark? Is it not a serious thing, for him to be rejected after attending a course of lectures, who, for several years before he experienced that advantage, had been a practising physician; had acquired to some extent the confidence of his neighborhood; and might, withal, be a person of honorable dispositions? Is it not better, that our schools should apply a prophylactic? especially when a dash of the faculty-pen would effectually meet the case? Three consequences result from the present regulation, which we deprecate. 1. It encourages many to engage in practice, whose enrolment as members of the profession, necessarily derogates from its scientific dignity. 2. It exposes society to all the evils which come from uneducated and incompetent medical guardians. 3. It tempts not a few to engage in the study, whose preparatory education, and means of future acquisition are deficient; and of course excludes an equal number, who might be adequately prepared. And here the subject expands upon us, and we shall pursue it somewhat further; premising that we write without consultation with our colleagues of the Institute, who are in no degree responsible for the opinions we express.

It is a popular and amiable idea (if amiability can be predicated of an opinion), that medical education and graduation should be brought within the reach of poor young men; and so it should, if it can at the same time be made such as to maintain the scientific dignity of the profession, and supply the community with physicians and surgeons who are thoroughly acquainted with all that is necessary to successful practice. These latter considerations impose limits to facility of graduation, which cannot be passed, without trampling under foot the demands of the science and the safety of society. All regulations, then, which encourage young men, unendowed with academic learning and the means of prolonged and ample medical study, to engage in the latter, are unjustifiable.

This mistaken beneficence to indigent young men, is a real (though undesigned) depredation on great interests which should never be overlooked. In a comparison of these, where no claims of justice are involved, those of the majority should be respected; especially, when by doing so the minority will not suffer. Such would be the fact in the present matter; for it cannot be doubted, that young men who have neither the preparatory education, nor the means of adequate connexion with medical schools, would do better for themselves, by directing their enterprize (often of the most laudable kind) upon pursuits, for which they are prepared, and for which their limited resources would be sufficient. It is certainly better to be a good merchant, mechanic, farmer or lawyer, than a bad doctor.

The late period of the sessions of our schools, at which students are permitted to enter, is an evil that should be corrected. In some of them, the limit is the 20th of November, in others the 30th. In all propriety it should be the second, supposing the session to open the first, Monday of that month. Why should more than the introductory week be allowed? If the session is too long, let it be shortened; but no argument can be offered for not *requiring* students to be present at the opening of the regular lectures. Are not those of the first fortnight as important as any? We may, rather, ask whether the student who fails to hear *them*, will not be crippled in his intellectual movements, throughout the whole course? How can he get on, understandingly, without the *data* which are laid down in the seventy-two lectures delivered in that fortnight?

But we must say a word on the early departure, not less than the late arrival of pupils. In every school of the United States, of which we know any thing touching this matter, numerous students go home, before the expiration of the session—unfortunately, *criminally* short as it is. We have repeatedly seen young men in the month of February descending the Ohio river, on their return from the schools of the east; and not less from our venerable alma mater, than the younger members of the sisterhood. From every school of the West, they begin to depart by the end of the second week in February, and sometimes earlier. The rate of desertion augments with the time, so that by the end of the third week, the class is in a state of rapid decomposition, leaving for the fourth but a nucleus, which by the last Saturday is reduced so low, that the delivery of a valedictory becomes a ridiculous formality. Thus, from late matriculation and early departure, the majority of every class lose from a fifth to a fourth of the session. But the mischief is not confined to them. It extends to the more ambitious, conscientious and persevering pupils, who are disturbed and agitated by the movements of their friends and room-mates; so that, as every observing professor knows, all intense application is at an end, from the time the breaking up begins. We grant that the professors of a medical school cannot, like an academic faculty, look after and see that its pupils are punctual in their attendance on the lectures; but it can be required, that all who intend to graduate, shall take with them at the close of the session, a ticket of dismission, as they receive one of admission when they enter. And why should not this be done? With a feeling bordering on sadness, we ask why it has not been done? Do the professors of our schools cherish no regard for the dignity or advancement of the noble profession confided to their guardianship? Do they think of nothing but the fees of tuition? Do they feel no responsibility to society? Will they continue to measure themselves by themselves, and look only to the *relative* attainments of their pupils; satisfied if they graduate none who fall beneath the low standard which harmonizes with an imperfect and superficial course of study? It may be said, that many young men are too poor to stay through the session; to which we reply, that all such had better not come, but devote themselves to other pursuits.

Let it not be said that if several of the facilities to matriculation and graduation in our schools, which now exist (but which we do not propose to enumerate at this time), were superseded by others, requiring higher preparatory attainment, longer professional study, and deeper scientific impregnation, there would be any deficiency of physicians. If it should a little diminish the aggregate number, it would increase the efficiency of each, and society would be better served, with less of that *downward* competition, which prevails not less among physicians than professors. The first and greatest effect of the new regulations would be, to invite to the study of medicine, those, and those only, who are well prepared by preliminary instruction, pecuniary means, talents and ambition, to make the profound and varied acquisitions in science, which are indispensable to the progressive improvement of the profession, and the safety and welfare of society. Many such are now excluded, because they see that superficial attainment, even without future study, may procure business; and not choosing to compete on such low ground, they turn to other pursuits. We *might* pursue this subject much further, and may resume it, at some convenient time. D.

